# Supplementary figures and images for: Netcombin: An algorithm for constructing optimal phylogenetic network from rooted triplets
Source: PLoS One. 2020 Sep 18;15(9):e0227842. doi: 10.1371/journal.pone.0227842 (PMC7500971; doi:10.1371/journal.pone.0227842)

$\vdash 0.0010$

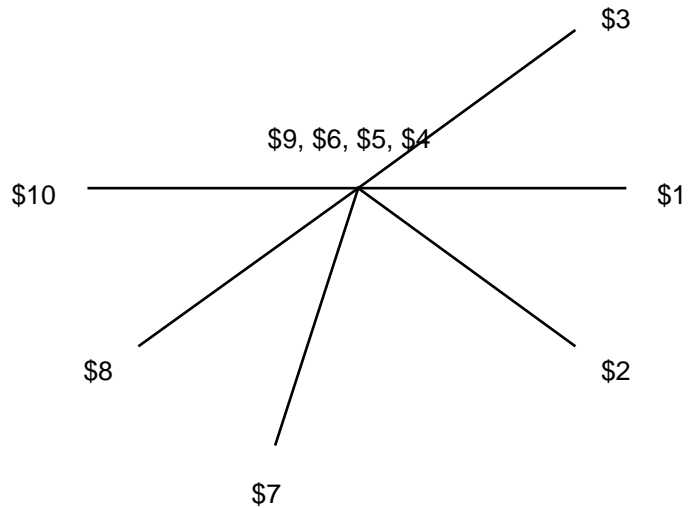

Supplement: S4 File — (ZIP) [file pone.0227842.s004.zip › SequenceData-Triplet/sequenceData-10-20-30-0-Triplet/length 100, numseq 10, rate 10^-7/1 seq.pdf]

H0.0010

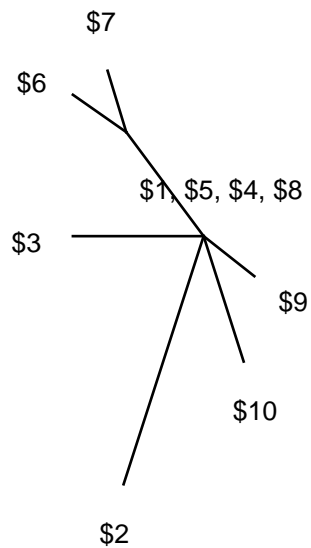

Supplement: S4 File — (ZIP) [file pone.0227842.s004.zip › SequenceData-Triplet/sequenceData-10-20-30-0-Triplet/length 100, numseq 10, rate 10^-7/10 seq.pdf]

$\vdash 0.0010$

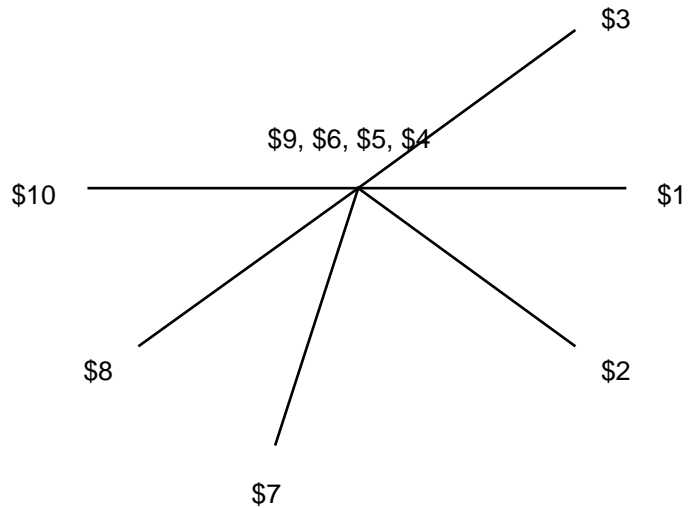

Supplement: S4 File — (ZIP) [file pone.0227842.s004.zip › SequenceData-Triplet/sequenceData-10-20-30-0-Triplet/length 100, numseq 10, rate 10^-7/2 seq.pdf]

$\vdash 0.0010$

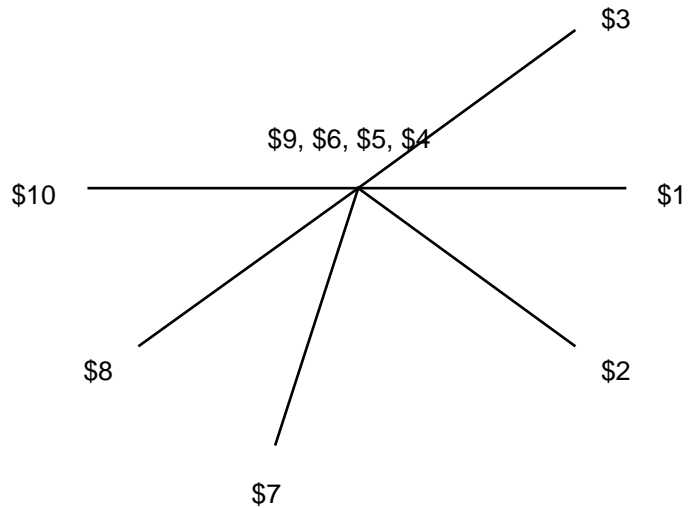

Supplement: S4 File — (ZIP) [file pone.0227842.s004.zip › SequenceData-Triplet/sequenceData-10-20-30-0-Triplet/length 100, numseq 10, rate 10^-7/3 seq.pdf]

$\vdash 0.0010$

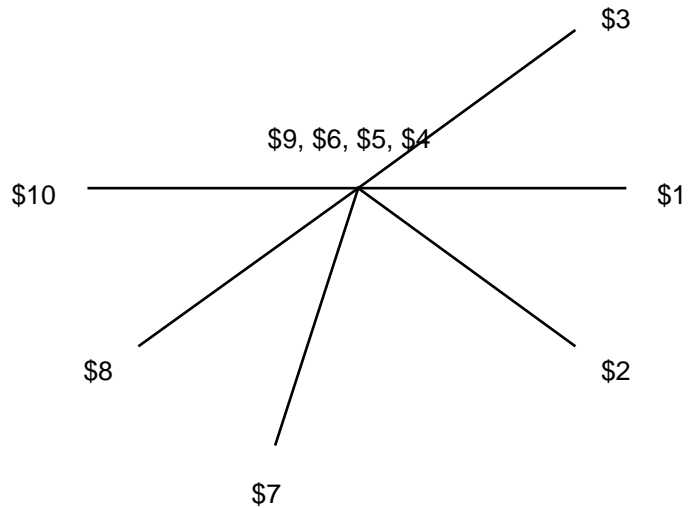

Supplement: S4 File — (ZIP) [file pone.0227842.s004.zip › SequenceData-Triplet/sequenceData-10-20-30-0-Triplet/length 100, numseq 10, rate 10^-7/4 seq.pdf]

$\vdash 0.0010$

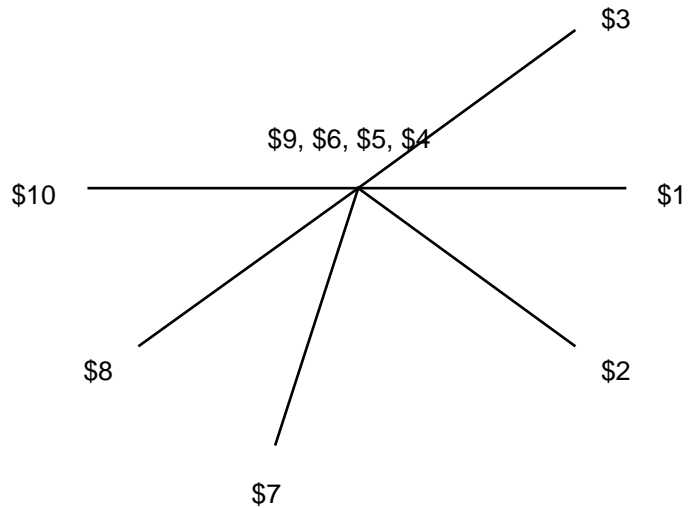

Supplement: S4 File — (ZIP) [file pone.0227842.s004.zip › SequenceData-Triplet/sequenceData-10-20-30-0-Triplet/length 100, numseq 10, rate 10^-7/5 seq.pdf]

H0.0010

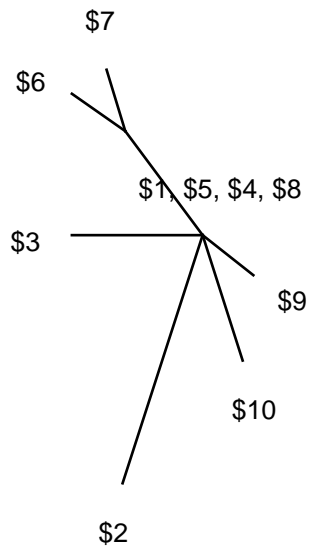

Supplement: S4 File — (ZIP) [file pone.0227842.s004.zip › SequenceData-Triplet/sequenceData-10-20-30-0-Triplet/length 100, numseq 10, rate 10^-7/6 seq.pdf]

H0.0010

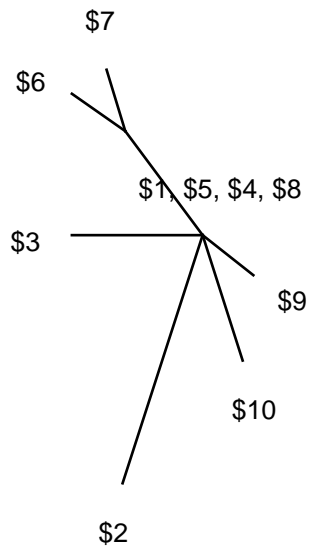

Supplement: S4 File — (ZIP) [file pone.0227842.s004.zip › SequenceData-Triplet/sequenceData-10-20-30-0-Triplet/length 100, numseq 10, rate 10^-7/7 seq.pdf]

H0.0010

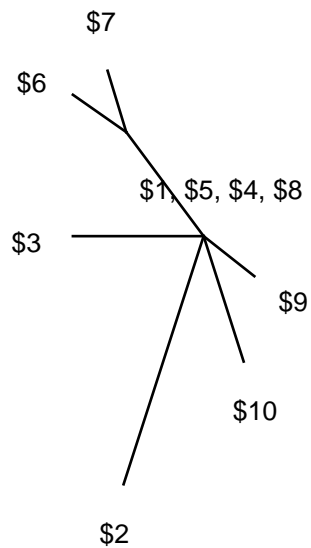

Supplement: S4 File — (ZIP) [file pone.0227842.s004.zip › SequenceData-Triplet/sequenceData-10-20-30-0-Triplet/length 100, numseq 10, rate 10^-7/8 seq.pdf]

H0.0010

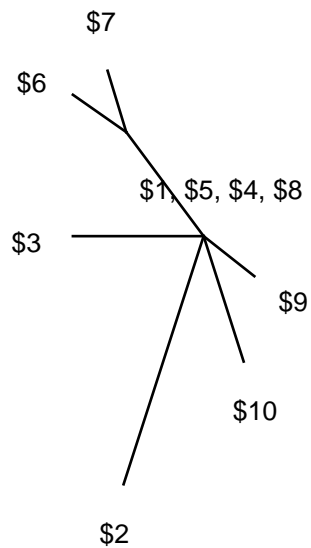

Supplement: S4 File — (ZIP) [file pone.0227842.s004.zip › SequenceData-Triplet/sequenceData-10-20-30-0-Triplet/length 100, numseq 10, rate 10^-7/9 seq.pdf]

H0.0010

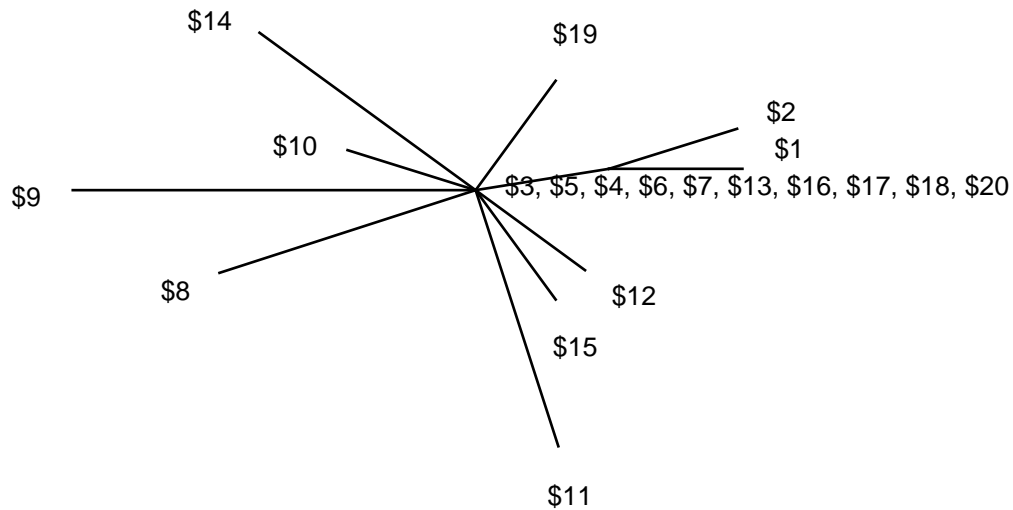

Supplement: S4 File — (ZIP) [file pone.0227842.s004.zip › SequenceData-Triplet/sequenceData-10-20-30-0-Triplet/length 100, numseq 20, rate 10^-7/1 seq.pdf]

H0.0010

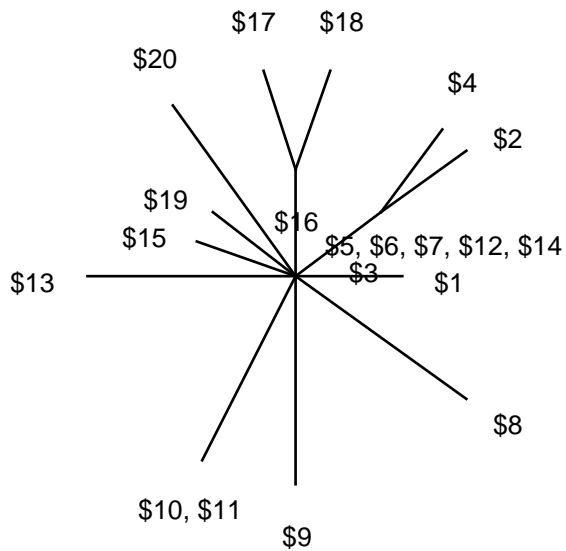

Supplement: S4 File — (ZIP) [file pone.0227842.s004.zip › SequenceData-Triplet/sequenceData-10-20-30-0-Triplet/length 100, numseq 20, rate 10^-7/10 seq.pdf]

H0.0010

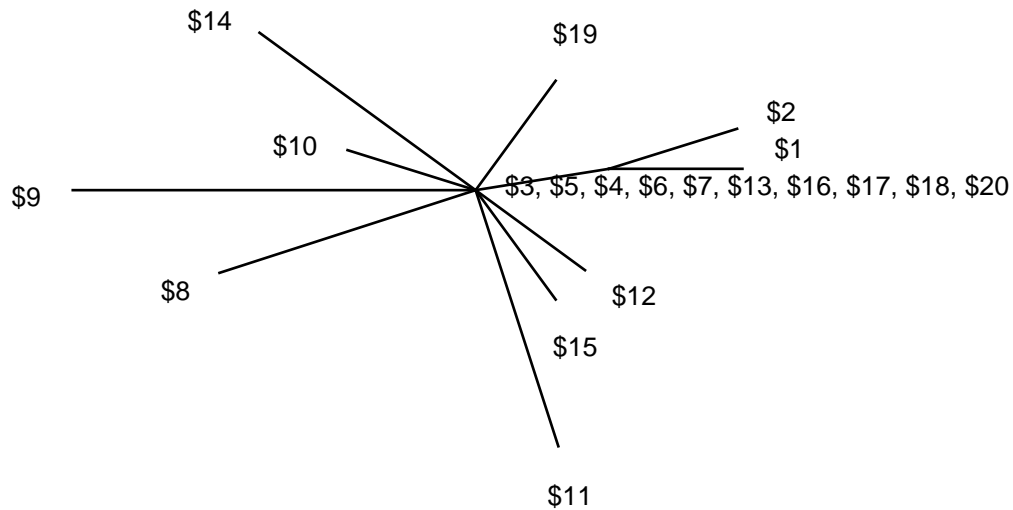

Supplement: S4 File — (ZIP) [file pone.0227842.s004.zip › SequenceData-Triplet/sequenceData-10-20-30-0-Triplet/length 100, numseq 20, rate 10^-7/2 seq.pdf]

H0.0010

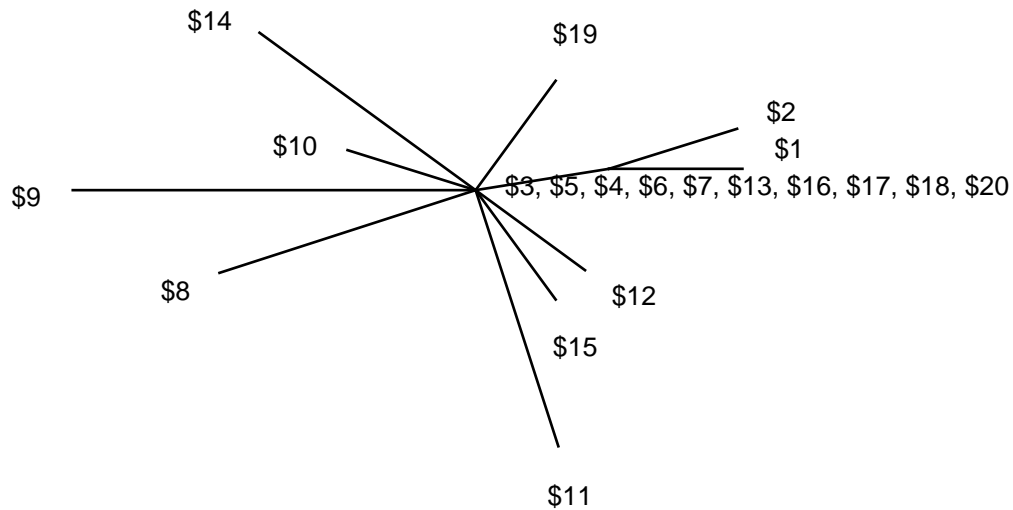

Supplement: S4 File — (ZIP) [file pone.0227842.s004.zip › SequenceData-Triplet/sequenceData-10-20-30-0-Triplet/length 100, numseq 20, rate 10^-7/3 seq.pdf]

H0.0010

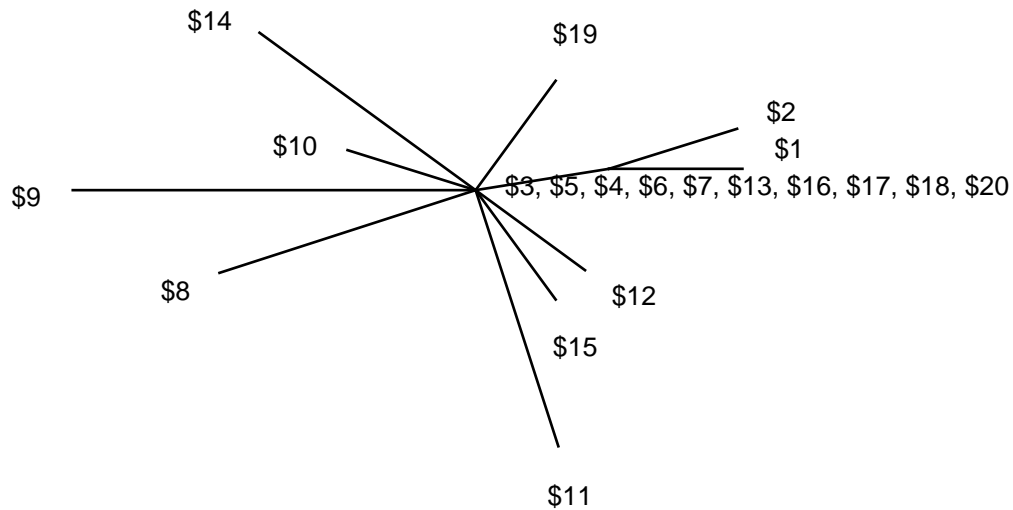

Supplement: S4 File — (ZIP) [file pone.0227842.s004.zip › SequenceData-Triplet/sequenceData-10-20-30-0-Triplet/length 100, numseq 20, rate 10^-7/4 seq.pdf]

H0.0010

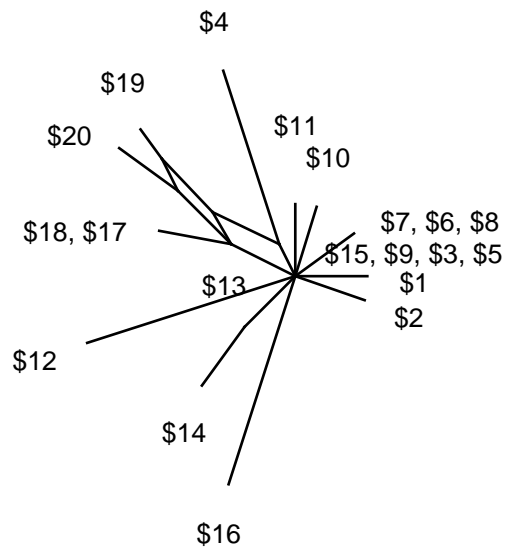

Supplement: S4 File — (ZIP) [file pone.0227842.s004.zip › SequenceData-Triplet/sequenceData-10-20-30-0-Triplet/length 100, numseq 20, rate 10^-7/5 seq.pdf]

H0.0010

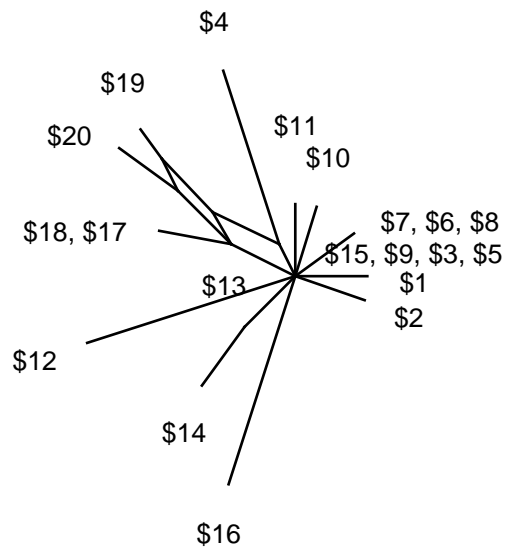

Supplement: S4 File — (ZIP) [file pone.0227842.s004.zip › SequenceData-Triplet/sequenceData-10-20-30-0-Triplet/length 100, numseq 20, rate 10^-7/6 seq.pdf]

H0.0010

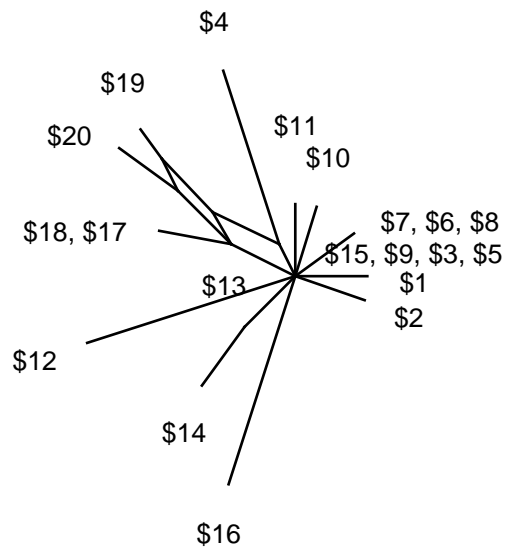

Supplement: S4 File — (ZIP) [file pone.0227842.s004.zip › SequenceData-Triplet/sequenceData-10-20-30-0-Triplet/length 100, numseq 20, rate 10^-7/7 seq.pdf]

H0.0010

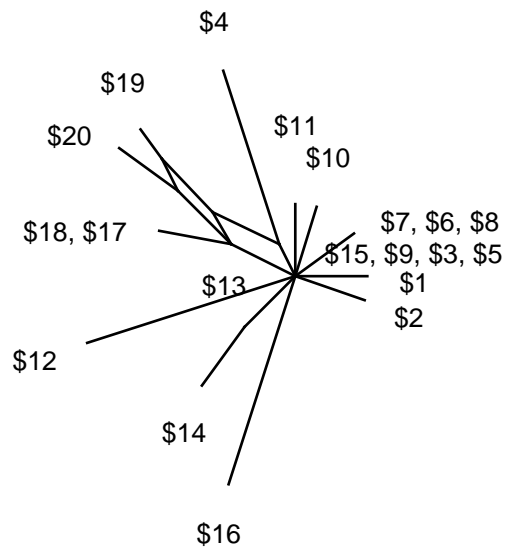

Supplement: S4 File — (ZIP) [file pone.0227842.s004.zip › SequenceData-Triplet/sequenceData-10-20-30-0-Triplet/length 100, numseq 20, rate 10^-7/8 seq.pdf]

H0.0010

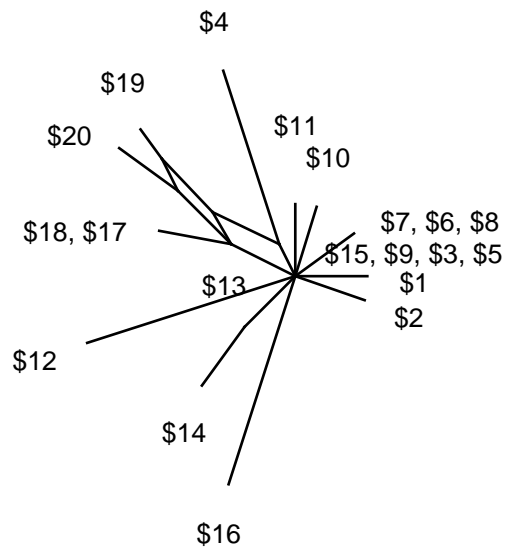

Supplement: S4 File — (ZIP) [file pone.0227842.s004.zip › SequenceData-Triplet/sequenceData-10-20-30-0-Triplet/length 100, numseq 20, rate 10^-7/9 seq.pdf]

H0.0010

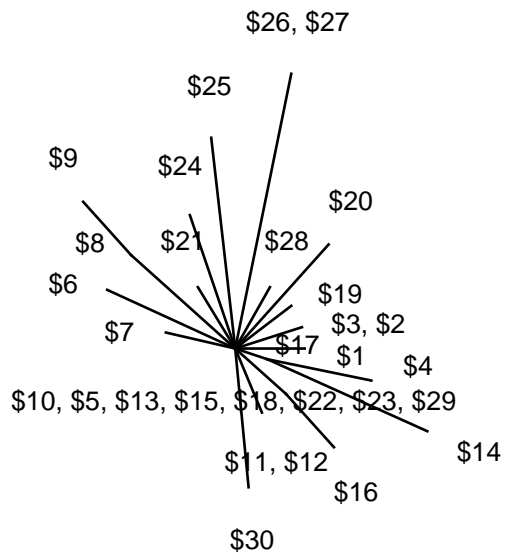

Supplement: S4 File — (ZIP) [file pone.0227842.s004.zip › SequenceData-Triplet/sequenceData-10-20-30-0-Triplet/length 100, numseq 30, rate 10^-7/1 seq.pdf]

H0.0010

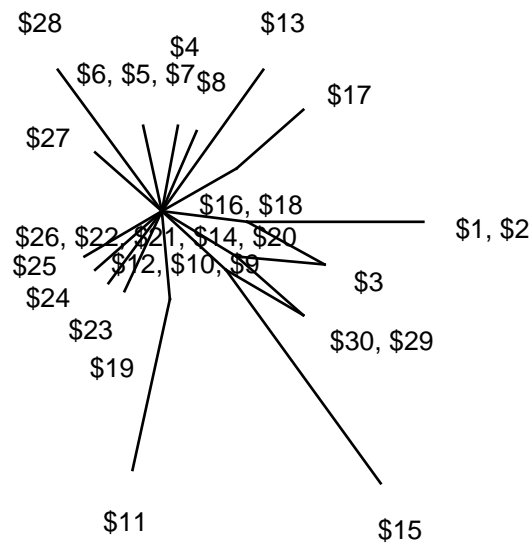

Supplement: S4 File — (ZIP) [file pone.0227842.s004.zip › SequenceData-Triplet/sequenceData-10-20-30-0-Triplet/length 100, numseq 30, rate 10^-7/10 seq.pdf]

H0.0010

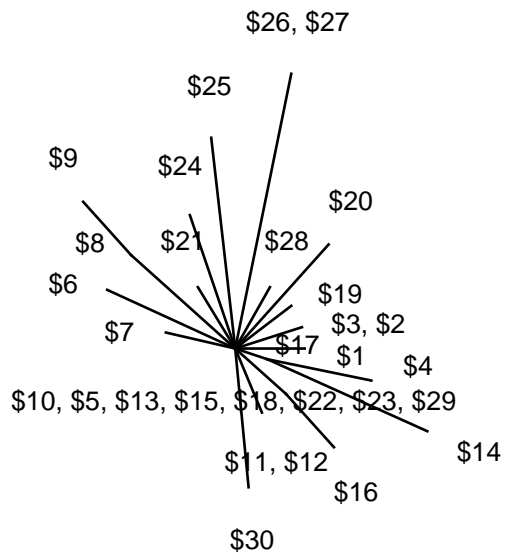

Supplement: S4 File — (ZIP) [file pone.0227842.s004.zip › SequenceData-Triplet/sequenceData-10-20-30-0-Triplet/length 100, numseq 30, rate 10^-7/2 seq.pdf]

H0.0010

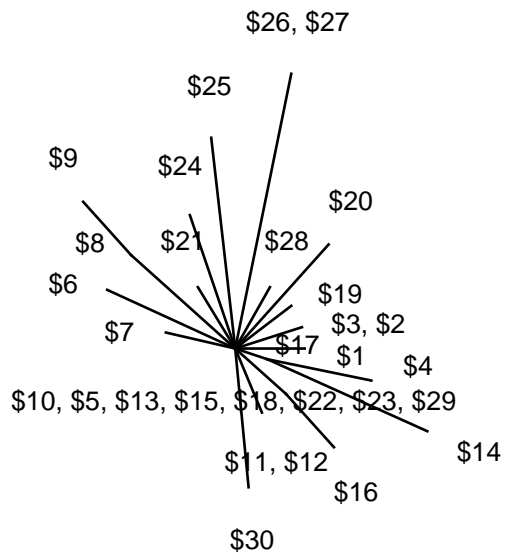

Supplement: S4 File — (ZIP) [file pone.0227842.s004.zip › SequenceData-Triplet/sequenceData-10-20-30-0-Triplet/length 100, numseq 30, rate 10^-7/3 seq.pdf]

0.01

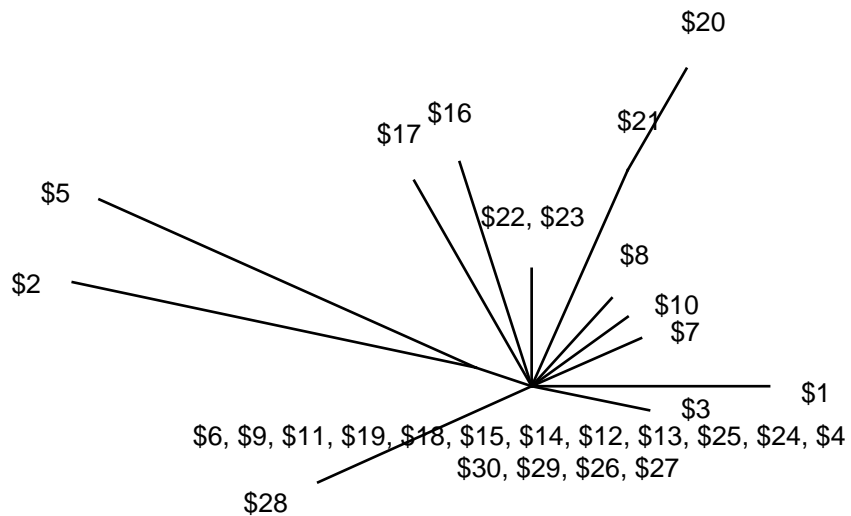

Supplement: S4 File — (ZIP) [file pone.0227842.s004.zip › SequenceData-Triplet/sequenceData-10-20-30-0-Triplet/length 100, numseq 30, rate 10^-7/4 seq.pdf]

0.01

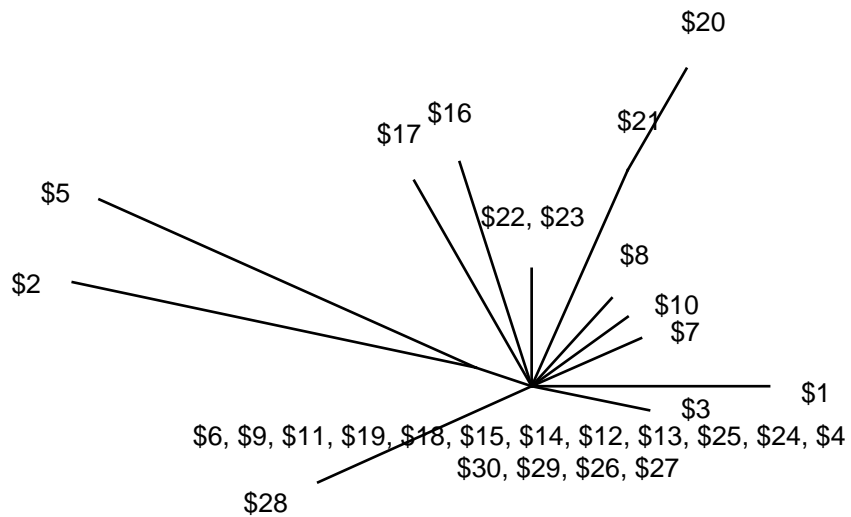

Supplement: S4 File — (ZIP) [file pone.0227842.s004.zip › SequenceData-Triplet/sequenceData-10-20-30-0-Triplet/length 100, numseq 30, rate 10^-7/5 seq.pdf]

H0.0010

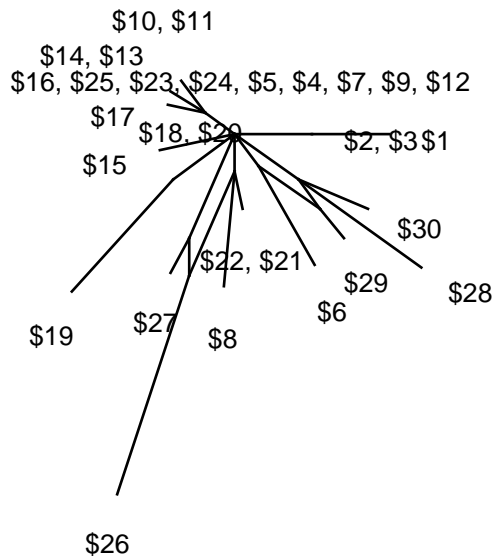

Supplement: S4 File — (ZIP) [file pone.0227842.s004.zip › SequenceData-Triplet/sequenceData-10-20-30-0-Triplet/length 100, numseq 30, rate 10^-7/6 seq.pdf]

H0.0010

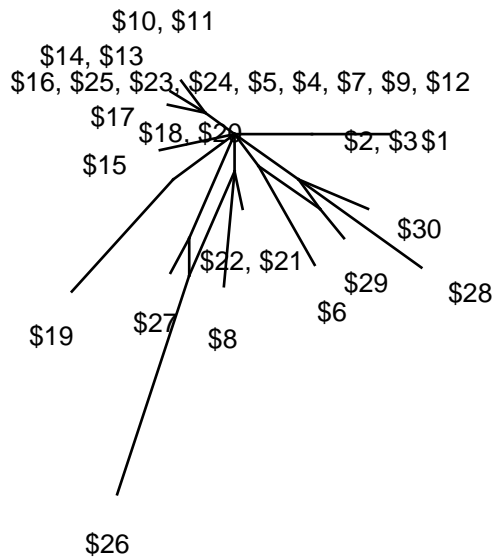

Supplement: S4 File — (ZIP) [file pone.0227842.s004.zip › SequenceData-Triplet/sequenceData-10-20-30-0-Triplet/length 100, numseq 30, rate 10^-7/7 seq.pdf]

H 0.0010

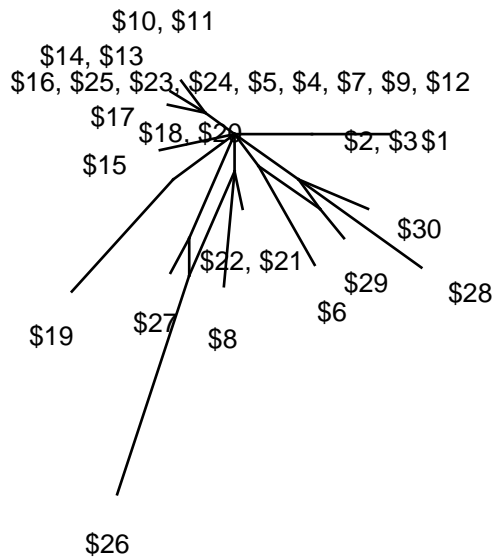

Supplement: S4 File — (ZIP) [file pone.0227842.s004.zip › SequenceData-Triplet/sequenceData-10-20-30-0-Triplet/length 100, numseq 30, rate 10^-7/8 seq.pdf]

H0.0010

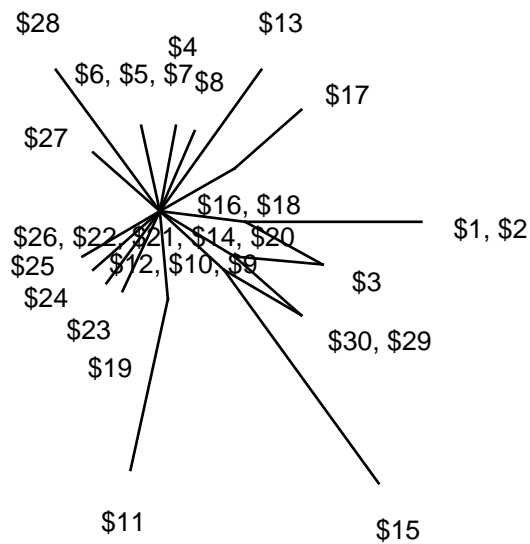

Supplement: S4 File — (ZIP) [file pone.0227842.s004.zip › SequenceData-Triplet/sequenceData-10-20-30-0-Triplet/length 100, numseq 30, rate 10^-7/9 seq.pdf]

H0.0010

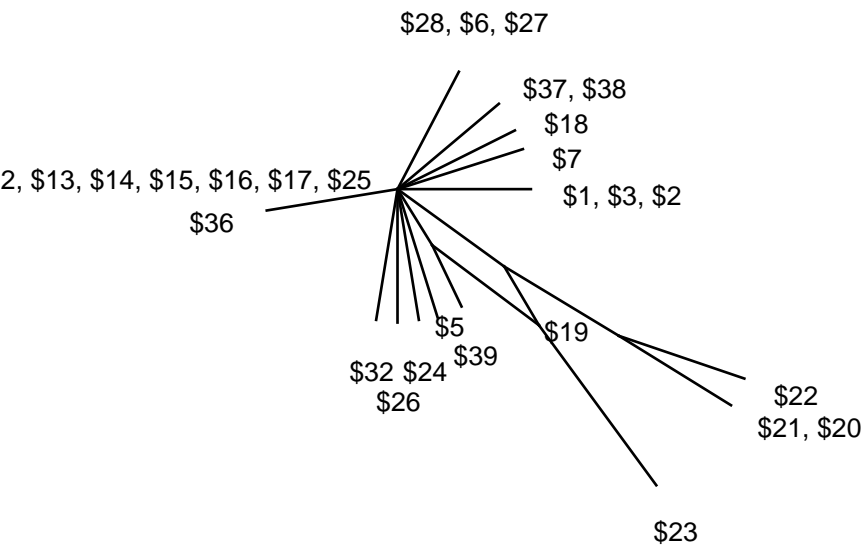

Supplement: S4 File — (ZIP) [file pone.0227842.s004.zip › SequenceData-Triplet/sequenceData-10-20-30-0-Triplet/length 100, numseq 40, rate 10^-7/1 seq.pdf]

H0.0010

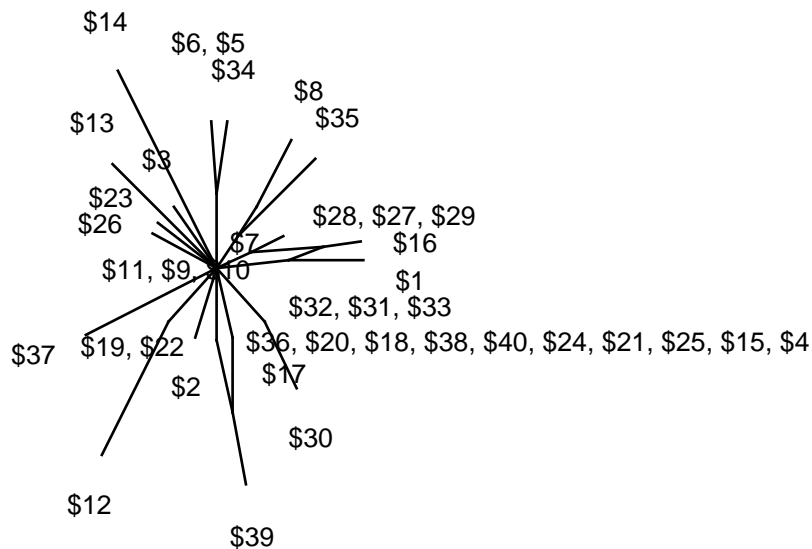

Supplement: S4 File — (ZIP) [file pone.0227842.s004.zip › SequenceData-Triplet/sequenceData-10-20-30-0-Triplet/length 100, numseq 40, rate 10^-7/10 seq.pdf]

0.01

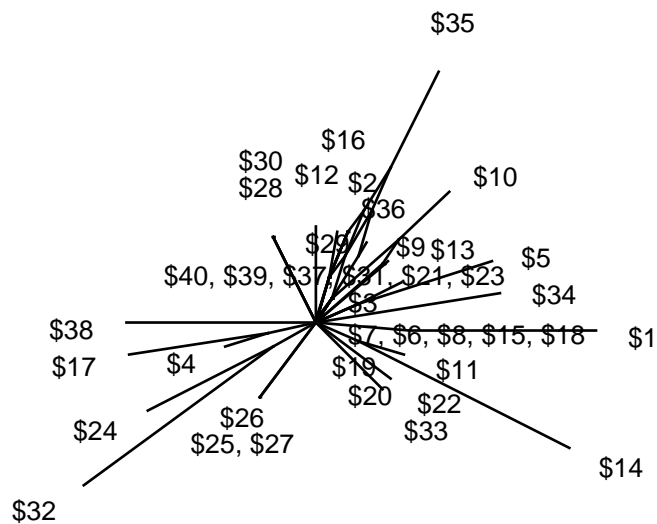

Supplement: S4 File — (ZIP) [file pone.0227842.s004.zip › SequenceData-Triplet/sequenceData-10-20-30-0-Triplet/length 100, numseq 40, rate 10^-7/2 seq.pdf]

0.01

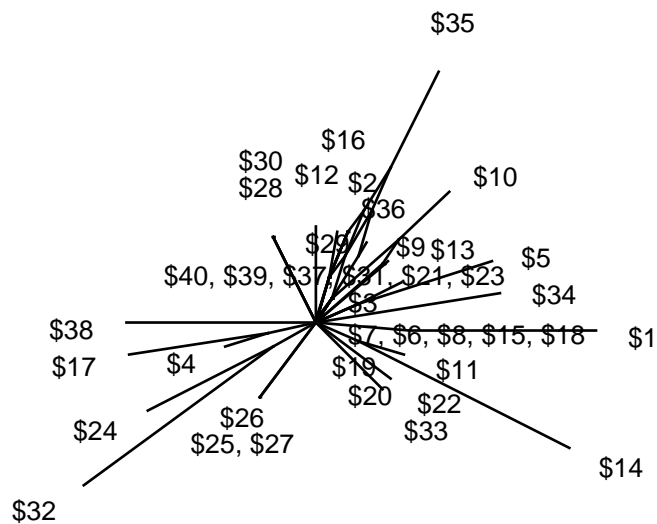

Supplement: S4 File — (ZIP) [file pone.0227842.s004.zip › SequenceData-Triplet/sequenceData-10-20-30-0-Triplet/length 100, numseq 40, rate 10^-7/3 seq.pdf]

H0.0010

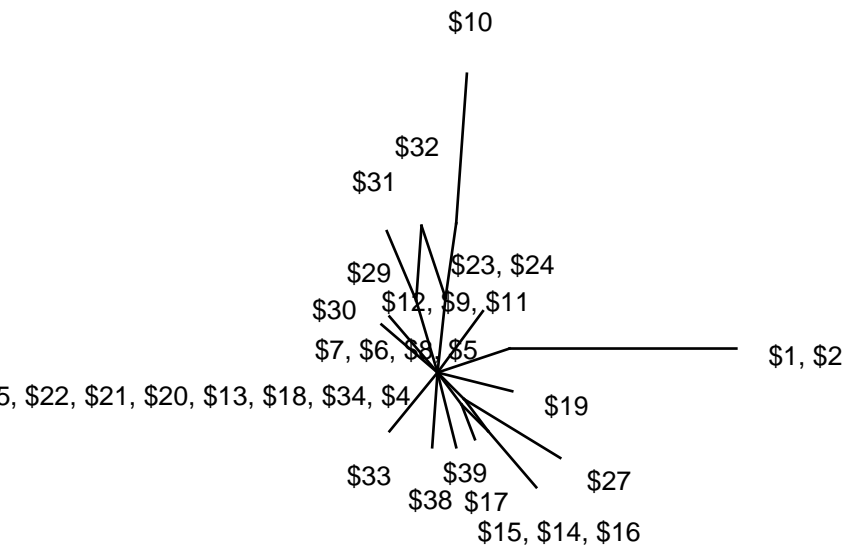

Supplement: S4 File — (ZIP) [file pone.0227842.s004.zip › SequenceData-Triplet/sequenceData-10-20-30-0-Triplet/length 100, numseq 40, rate 10^-7/4 seq.pdf]

H0.0010

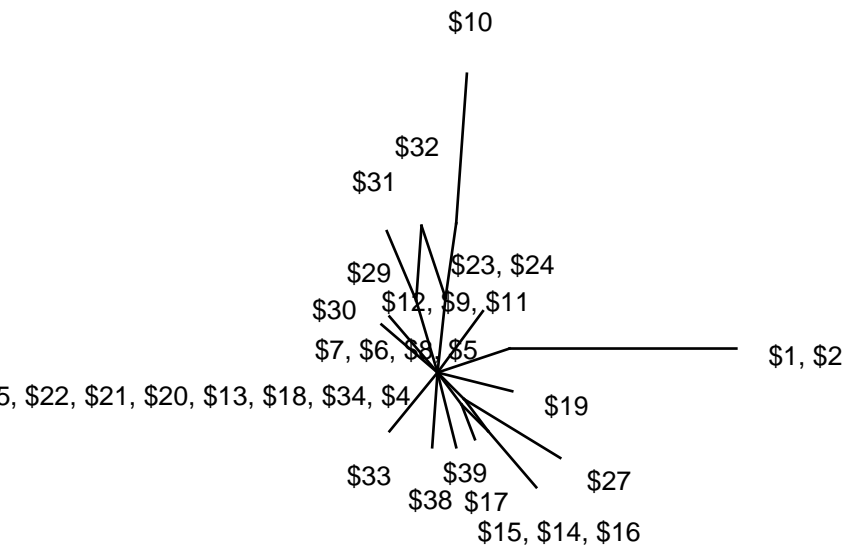

Supplement: S4 File — (ZIP) [file pone.0227842.s004.zip › SequenceData-Triplet/sequenceData-10-20-30-0-Triplet/length 100, numseq 40, rate 10^-7/5 seq.pdf]

H0.0010

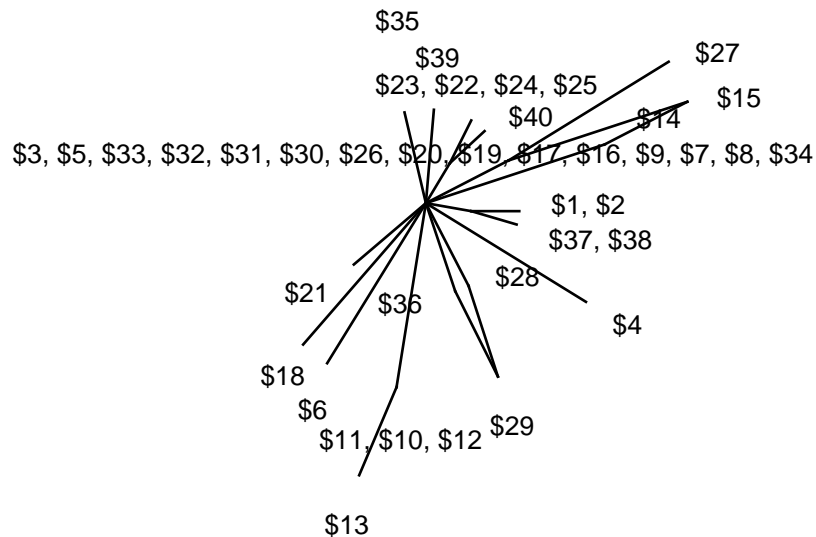

Supplement: S4 File — (ZIP) [file pone.0227842.s004.zip › SequenceData-Triplet/sequenceData-10-20-30-0-Triplet/length 100, numseq 40, rate 10^-7/6 seq.pdf]

H0.0010

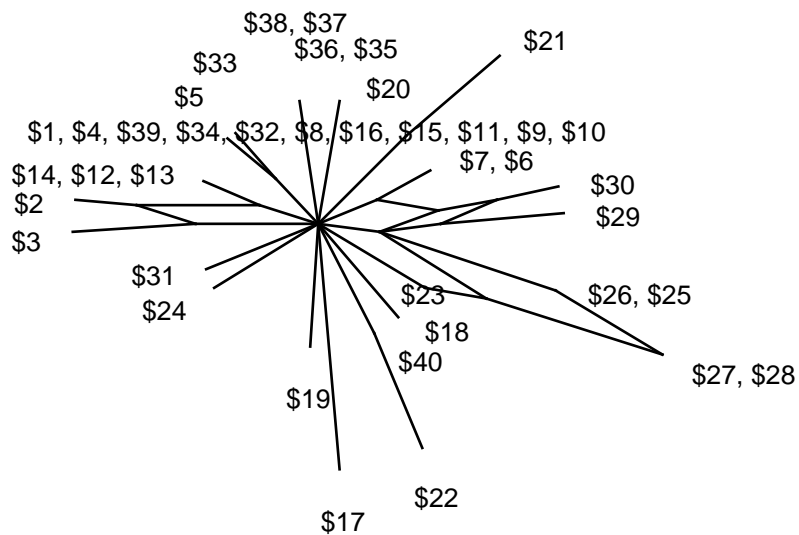

Supplement: S4 File — (ZIP) [file pone.0227842.s004.zip › SequenceData-Triplet/sequenceData-10-20-30-0-Triplet/length 100, numseq 40, rate 10^-7/7 seq.pdf]

H0.0010

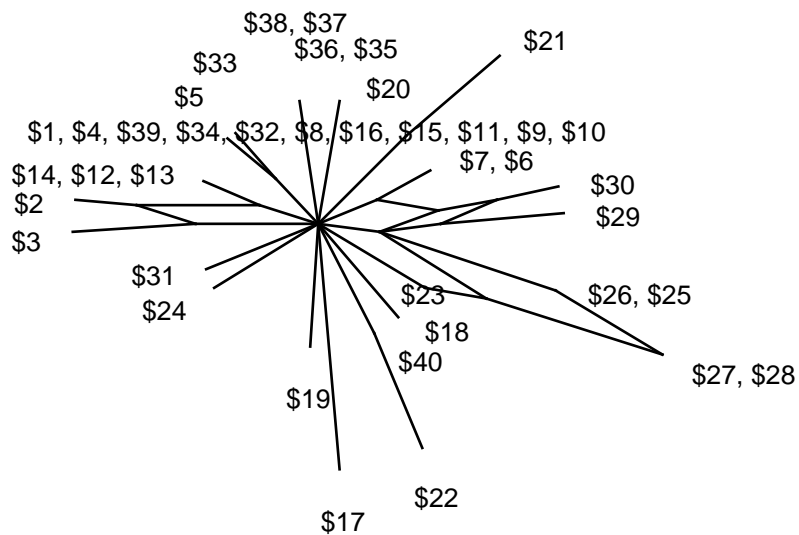

Supplement: S4 File — (ZIP) [file pone.0227842.s004.zip › SequenceData-Triplet/sequenceData-10-20-30-0-Triplet/length 100, numseq 40, rate 10^-7/8 seq.pdf]
